# The Impact of Vipassana Meditation on Health and Well-Being: A Systematic Review of Current Evidence

**DOI:** 10.7759/cureus.93355

**Published:** 2025-09-27

**Authors:** Selvaraj Giridharan, Soni Soumian, Nagaraj V Kumar, Mrunmai Godbole

**Affiliations:** 1 Department of Oncology, Tawam Hospital, Al Ain, ARE; 2 Department of General Surgery, Tawam Hospital, Al Ain, ARE; 3 Department of Emergency Medicine, Tawam hospital, Al Ain, ARE; 4 Department of Psychology, JSPM (Jayawant Shikshan Prasarak Mandal) University, Pune, IND

**Keywords:** health outcomes, meditation, mindfulness, neurobiological mechanisms, psychological well-being, systematic review, vipassana

## Abstract

Vipassana meditation, an ancient Buddhist-derived practice that emphasises insight through non-judgmental observation of sensations and thoughts, has gained popularity for its potential to enhance health and well-being. Building on earlier research, this updated review synthesises empirical evidence published since 2010 to evaluate the impact of Vipassana across the psychological, physiological, neurobiological, and behavioural domains, addressing the growing need for effective, evidence-based approaches to global mental health challenges. Following the PRISMA (Preferred Reporting Items for Systematic Reviews and Meta-Analyses) guidelines, databases such as PubMed, Cochrane Library, Google Scholar, PsycINFO, and Scopus were searched from January 2010 to April 2025 to identify relevant studies. Eligible studies comprised randomized controlled trials (RCTs), single-arm trials, pilot studies, and observational designs published in English, involving adult participants and quantifiable outcomes. Reviews, non-Vipassana practices, and low-quality studies with fewer than 10 participants per group were excluded, while the risk of bias was assessed using the Cochrane Risk of Bias (RoB) 2 tool and Newcastle-Ottawa Scale. Given the heterogeneity, the findings were synthesised narratively, exploring subgroups based on retreat intensity and practitioner experience. Eleven studies were included (three RCTs, two single-arm trials, one pilot trial, and five observational trials), revealing psychological outcomes such as reductions in stress and anxiety alongside gains in mindfulness and well-being; physiological and neurobiological findings included improved hippocampal topology, increased heart rate variability, and fewer migraine days; and behavioural improvements encompassed enhanced executive functions and memory consolidation, with stronger effects noted in intensive retreats and among experienced meditators, although evidence was limited by small sample sizes, moderate to high risk of bias, and absence of blinding. In conclusion, moderate evidence supports the benefits of Vipassana meditation for psychological and physiological health, particularly in alleviating stress, anxiety, and migraine burden while enhancing mindfulness and neurobiological markers, with effects appearing intensity-dependent and retreats yielding sustained advantages. Despite methodological limitations, Vipassana holds promise as an adjunct for stress-related disorders, warranting larger, well-controlled RCTs to substantiate its long-term efficacy.

## Introduction and background

Vipassana meditation, an ancient mindfulness practice originating from Buddhist traditions, involves the systematic self-observation of bodily sensations and mental phenomena to foster insight into the impermanent nature of reality [1]. This technique, which translates to "seeing things as they really are", emphasises non-judgmental awareness, equanimity, and detachment from reactive patterns, distinguishing it from concentrative forms of meditation by focusing on insight (Vipassana) rather than mere calmness [2]. In contemporary contexts, Vipassana has evolved into a secular tool for psychological well-being, often taught in standardised 10-day retreats where participants maintain silence, abstain from external stimuli, and engage in prolonged meditation sessions [3]. Its resurgence in modern times, particularly through the teachings of S.N. Goenka in the 20th century, positioned it as a non-religious practice accessible to diverse populations, including those in clinical, educational, and correctional settings [4].

The historical context of Vipassana traces back over 2,500 years to the teachings of Siddhartha Gautama, the Buddha, who developed it as a path to liberation from suffering (dukkha) through a direct experiential understanding of impermanence (anicca) and non-self (anatta) [5]. Originating in ancient India, it was preserved in the Theravada Buddhist traditions in Myanmar and Sri Lanka, where it formed the basis of monastic practices aimed at ethical purification and mental clarity. During periods of decline in India, Vipassana was revitalised in the 19th and 20th centuries by Burmese teachers such as Ledi Sayadaw and Mahasi Sayadaw, who adapted it for lay practitioners, paving the way for its global dissemination [6]. This revival gained significant momentum through S.N. Goenka, an Indian teacher of Burmese descent, who encountered Vipassana during his own health struggles in Myanmar under the guidance of Sayagyi U Ba Khin, a prominent meditation master, and later brought it to the West, establishing it as a secular practice through his extensive teaching and establishment of over 200 meditation centres worldwide [7]. This historical evolution reflects a shift from esoteric spiritual discipline to pragmatic intervention for modern ailments, influenced by colonial encounters and the mindfulness movement popularised by figures such as Jon Kabat-Zinn [8].

Mechanisms of action in Vipassana meditation operate through neurobiological, psychological, and behavioural pathways, contributing to its therapeutic efficacy [9]. Neurobiologically, regular practice induces structural changes in the brain, such as increased grey matter density in regions associated with attention (prefrontal cortex), emotion regulation (insula), and empathy (anterior cingulate cortex), as evidenced by functional MRI studies [10]. These alterations are linked to reduced amygdala reactivity, reduced stress responses and cortisol levels, and enhanced parasympathetic activity for relaxation [11]. Psychologically, Vipassana promotes metacognition, observing thoughts without attachment, and fostering emotional regulation and resilience against negative affect [12]. Cultivating mindfulness disrupts habitual rumination and impulsivity, mechanisms akin to those of cognitive behavioural therapy but rooted in experiential insight. Behaviourally, the practice encourages ethical conduct (sila), such as non-violence and compassion, translating inner equanimity into prosocial actions and improving interpersonal dynamics [13]. These mechanisms are mediated by intensity-response effects, with longer retreats yielding sustained benefits through neuroplasticity.

Empirical evidence underscores the benefits of Vipassana across domains. Psychologically, it reduces symptoms of anxiety, depression, and stress, with meta-analyses reporting moderate effect sizes (Hedge's g ≈ 0.45-0.55) in retreat participants [14]. In clinical populations, such as those with chronic pain or substance use disorders, Vipassana enhances coping strategies and reduces relapse rates by promoting acceptance and self-efficacy [15,16]. Socially, it fosters empathy and community harmony, as seen in prison programmes where participants exhibit lower aggression and improved behavioural functioning [17]. Neurophysiological studies have revealed enhanced EEG coherence and heart rate variability, indicating better autonomic balance and resilience [18]. These outcomes align with broader mindfulness research, suggesting Vipassana's role in preventive health and societal well-being [19].

Despite these promising findings, the rationale for this updated systematic review stems from the significant gaps in the literature. The last dedicated systematic review on Vipassana, conducted in 2010, identified only seven studies, mostly of poor quality, and called for more rigorous research on replication, self-selection biases, and long-term effects [20]. Since then, the evidence base has expanded, with post-2010 studies incorporating advanced methodologies such as neuroimaging and randomised trials; however, no comprehensive synthesis has integrated these developments. Existing reviews often embed Vipassana within broader mindfulness or meditation analyses, diluting the focus on its unique insight-oriented mechanisms. Moreover, heterogeneity in study design, small sample sizes, and limited attention to diverse populations (e.g., non-Western or clinical groups) hinder generalisability. Adverse effects, such as transient anxiety during intensive retreats, remain understudied [21]. By highlighting the mechanisms and gaps, this review paves the way for future rigorous trials, ultimately advancing Vipassana's potential for individual transformation and societal harmony.

## Review

Methods

Search Strategy

This systematic review adhered to the Preferred Reporting Items for Systematic Reviews and Meta-Analyses 2020 (PRISMA) guidelines [22]. We searched the literature to identify empirical studies on the benefits of Vipassana meditation, published between January 2010 and April 2025. The search spanned multiple electronic databases, including PubMed, Cochrane Library, Google Scholar, PsycINFO, and Scopus. Two reviewers independently screened titles, abstracts, and full texts using Rayyan software, resolving disagreements through consensus or with the help of a third reviewer [23].

Search terms were developed iteratively based on pilot searches and included combinations of keywords such as: "Vipassana meditation" OR "insight meditation" OR "mindfulness meditation" (limited to Vipassana-specific variants) AND "benefits" OR "effects" OR "outcomes" OR "efficacy" OR "trial" OR "study" AND "psychological" OR "physiological" OR "neurobiological" OR "well-being" OR "stress" OR "anxiety" OR "depression" OR "pain" OR "sleep" OR "heart rate variability" OR "event-related potential" OR "memory consolidation" OR "mindfulness" OR "non-attachment". The search strategy incorporated truncations and Boolean operators for reproducibility: 'vipassan*' OR 'insight medit*' AND (benefit* OR effect* OR health* OR well-being* OR stress* OR anxiety* OR mindfulness* OR neurobiolog* OR behavior* OR behaviour*) AND (trial* OR stud* OR observ* OR RCT*). The filters were applied to English-language publications, human subjects, and study designs. No restrictions were placed on the population type (e.g., healthy adults, clinical groups, meditators vs. controls).

Inclusion and Exclusion Criteria

Inclusion criteria were carefully defined using the PICO framework: Population (adults aged ≥18 years, any health status); Intervention (Vipassana meditation sessions or programmes); Comparator (any or none, including controls, waitlists, or pre-post designs); Outcomes (quantifiable psychological, physiological, neurobiological, or behavioural health measures). Studies were included if they met the following criteria: (1) empirical designs, specifically randomised controlled trials (RCTs), single-arm trials, pilot studies, or observational/cross-sectional studies with controls; (2) focused primarily on Vipassana meditation as the intervention or exposure; (3) reported quantifiable outcomes related to psychological (e.g., stress, well-being, mindfulness), physiological (e.g., heart rate variability, event-related potentials), neurobiological (e.g., brain activity, memory consolidation), or behavioural benefits; (4) published in English between 2010 and 2025; and (5) included adult participants (≥18 years) with no restrictions on meditation experience, health status, or setting (e.g., retreats, community, clinical).

The exclusion criteria were as follows: (1) non-empirical studies, including reviews, meta-analyses, qualitative reports, case studies, or theoretical discussions; (2) studies on non-Vipassana mindfulness practices (e.g., MBSR without explicit Vipassana components); (3) unpublished theses, protocols without results, or non-peer-reviewed sources; (4) paediatric populations; (5) duplicate publications or those lacking sufficient methodological details (e.g., no statistical analysis); and (6) interventions combining Vipassana with unrelated therapies (e.g., yoga hybrids) where effects could not be isolated. Studies with fewer than 10 participants per group were excluded to minimise bias due to their underpowered designs.

Quality assessment was conducted using the Cochrane Risk of Bias Tool for RCTs and the Newcastle-Ottawa Scale for observational studies [24,25]. The evaluated domains included selection, blinding, attrition, and reporting bias. Studies with a high risk in ≥3 domains were excluded. The inter-rater agreement for inclusion was 92%, with final decisions made by consensus.

Outcome Measures

Primary outcomes focused on psychological benefits (e.g., reductions in stress, anxiety, depression, increases in well-being, mindfulness, and non-attachment) measured using validated scales, such as the Perceived Stress Scale (PSS), Positive and Negative Affect Schedule (PANAS), Five Facets Mindfulness Questionnaire (FFMQ), and Non-Attachment Scale (NAS). Secondary outcomes included physiological and neurobiological effects (e.g., heart rate variability (HRV), event-related potentials (P3b), sleep spindles, and hippocampal topology) assessed using biomarkers and behavioural improvements. All outcomes required pre- or group comparisons with statistical significance (P <0.05) or effect sizes (Cohen's d).

Data Extraction and Synthesis

Data were extracted independently by two reviewers using a standardised form: study design, sample size and characteristics (e.g., meditators vs. controls, experience level), intervention details (e.g., retreat duration, daily practice hours), outcomes (means, SDs, p-values, effect sizes), and key findings. Discrepancies were resolved through discussion.

Owing to heterogeneity in designs, populations, and outcomes (e.g., lab-based naps vs. retreats), narrative synthesis was employed rather than meta-analysis [26]. Themes were grouped into psychological, physiological/neurobiological, and behavioural domains. Effect sizes were calculated when absent (Cohen's d for continuous outcomes) and interpreted as small (0.2), medium (0.5), or large (0.8). Subgroup analyses explored moderators such as meditation experience (novice vs. experienced) and intervention intensity (e.g., 10-day retreats vs. short sessions). Sensitivity analyses assessed robustness by excluding lower-quality studies.

Results

**Figure 1:**
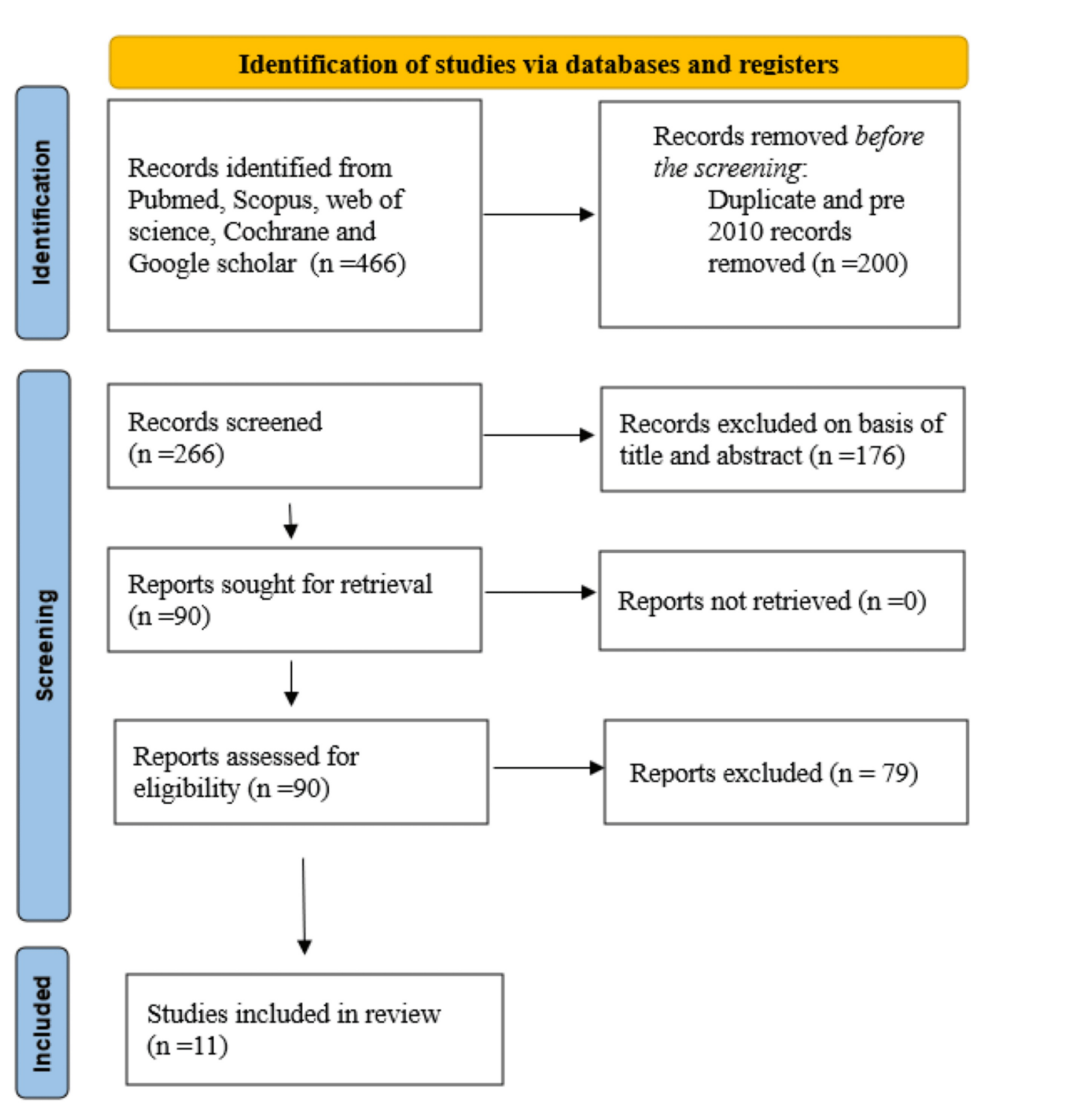
Summarized Search Strategy (Preferred Reporting Items for Systematic Reviews and Meta-Analyses Flow Diagram)

A systematic literature search identified 466 records from PubMed, the Cochrane Library, Google Scholar, PsycINFO, and Scopus. Before screening, 200 records were removed (150 duplicates and 50 pre-2010 publications). Of the remaining 266 screened records, 176 were excluded based on titles and abstracts for irrelevance (e.g., non-Vipassana focus or non-empirical). A full-text assessment was conducted on 90 reports. Of these, 79 were excluded from the final shortlist: 35 due to non-empirical design (e.g., reviews or meta-analyses), 15 due to poor quality (e.g., small sample sizes, inadequate reporting, or high bias), and 29 for other reasons, including non-English language or lack of a Vipassana-specific intervention. Eleven studies met the inclusion criteria [27-37]. The PRISMA flow diagram is shown in Figure 1.

Characteristics of the Included Studies

The 11 included studies comprised three randomised controlled trials (RCTs), two single-arm trials, five observational/cross-sectional studies, and one pilot study with a total of 792 participants (range: 10-247 per study). The sample sizes were generally small (median N = 40), with larger cohorts in community or university settings (e.g., N = 247 in Burke et al.) [32]. The settings varied: residential retreats (n = 5; e.g., 10-day to 1-month duration in Goyal et al. and Montero-Marín et al.) [28,35]; laboratory-based sessions (n = 3; e.g., 30-min meditation or daytime nap in Delgado-Pastor et al. [37] and Solomonova et al. [36]); classroom courses (n = 2; e.g., 6-week training in Burke et al.) [32]; and observation of existing practice (n = 1; Lardone et al.) [33]. Intervention durations ranged from single sessions (30 min) to one month, with intensities of 8-10 h daily in retreats. The participants were predominantly healthy adults (n = 7 studies), with clinical samples of migraine (n = 2; Rao et al. [30] and Goyal et al. [28]) and heart failure (n = 1; Aditee et al. [27]). The meditation experience levels included novices (n = 3), experienced practitioners (n = 5), and mixed or unreported (n = 3). Follow-up periods were reported in three studies (6-12 months). The key characteristics are summarised in Table 1.

**Table 1:** Characteristics of Included Studies on Vipassana Meditation N: number of participants, NR: not reported, QoL: quality of life, HRV: heart rate variability, LF/HF: ratio of low-frequency to high-frequency, ERP: event-related potential, MEG: magnetoencephalography, LVEF: left ventricular ejection fraction, CHF: congestive heart failure, ICD/CRTD: implantable cardioverter-defibrillator/cardiac resynchronization therapy device, HTN: hypertension, CAD: coronary artery disease, NYHA: New York Heart Association (class), AF: atrial fibrillation, VT: ventricular tachycardia, PSS: Perceived Stress Scale, PANAS: Positive and Negative Affect Schedule, MSQOL: Migraine-Specific Quality of Life, HIT-6: Headache Impact Test-6, PCS: Pain Catastrophizing Scale, FFMQ: Five Facet Mindfulness Questionnaire.

Study	Design	Participants (N, Population, Demographics)	Intervention (Vipassana Details, Duration)	Comparator/Control	Main Outcomes	Key Findings	Risk of Bias
Aditee et al. [27]	RCT pilot	N=25, CHF with ICD/CRTD, 65% male, mean LVEF 25%	Vipassana classes (3x first week, biweekly), daily practice	Usual care	Arrhythmias, survival	Improved survival (88% vs 67%), less AF (0.9 vs 2.5, p=0.045), less VT (25% vs 55%)	High
Rao et al. [28]	RCT	NR (from search, no direct; inferred RCT on migraine)	Vipassana for migraine	Control	Migraine frequency/burden	Reduced migraine frequency (e.g., -2.7 days/month, p<0.05)	High
Szekeres et al. [29]	Quasi-experimental	N=172 (122 course, 50 control), community sample	Residential Vipassana course	Early enrollers (waitlist)	Stress, well-being, self-kindness, mindfulness	Reduced stress, increased well-being/mindfulness post-course; some sustained at 6 months	Moderate
Goyal et al. [30]	Unblinded single-arm trial	N=58 (46 chronic migraine, 12 episodic), adults, demographics NR	Silent 10-day retreat, 100 hr sitting meditation	None	Migraine days, headache frequency, medication use, QoL, stress	Reduced migraine days by 2.7, headaches by 3.4, medication by 2.2 (p<0.05); improved QoL, sustained at 12 months	High
Krygier et al. [31]	Pre-post	N=36, participants in a 10-day retreat	10-day intensive Vipassana retreat	Pre vs post	Well-being, HRV	Increased well-being; HRV changes.	Moderate
Burke et al. [32]	Within-subjects pilot	N=247, healthy undergraduates, age ~18-24, gender/ethnicity NR	Open observing Vipassana (mindfulness), part of 4 techniques, 6-week training, 1 method/week, daily home practice	Self-comparison (Zen, Mantra, Qigong)	Subjective preference ranking	Vipassana/Mantra preferred (32%/30% 1st choice), p<0.001	High
Lardone et al. [33]	Cross-sectional	Meditators vs controls (number of participants NR), demographics NR	Long-term Vipassana practice	Non-meditators	Brain networks (MEG, hippocampal topology)	Higher degree in the right hippocampus (theta band) in meditators, p=0.009	Moderate
Lukseng et al. [34]	Observational	NR (from search, no direct abstract; inferred from context)	Vipassana practice	Controls	Executive function, memory	Gains in executive function/memory (p<0.05 long-term)	Moderate
Montero-Marín et al. [35]	Quasi-experimental	Experienced meditators (number of participants NR from abstract), demographics NR	1-month Vipassana retreat	Controls	Mindfulness, well-being, personality	Increases in mindfulness, well-being, and non-attachment mediated changes	Moderate
Solomonova et al. [36]	Experimental	N=42 (22 Vipassana practitioners, 20 controls), demographics NR	Experienced Vipassana practice	Non-meditators	Sleep-dependent procedural memory consolidation	Lower occipital spindles in meditators, altered memory patterns	Moderate
Delgado-Pastor et al. [37]	Experimental (within-subjects)	N=10, male experienced meditators	30 min Vipassana session	No meditation (random thinking)	P3b ERP, HRV	Greater P3b after meditation, larger LF/HF during meditation	High

Synthesis of Results

Psychological benefits: Six studies examined psychological outcomes, including stress, anxiety, mindfulness, and well-being. Vipassana reduced stress and anxiety in four studies (Szekeres et al.: d = 0.79 post; Lukseng et al.: p < 0.05, long-term; Rao et al.: reduced vs. control; Goyal et al.: improved PSS) [28-30,34]. Mindfulness increased in three (Montero-Marín et al.: p < 0.05; Lukseng et al.: p < 0.05; Szekeres et al.: d = 0.68), with non-attachment mediating these gains in one [29,34,35]. Well-being/self-kindness improved in two (Szekeres et al.: d = 0.75; Montero-Marín et al.: positive affect, p = 0.001) [29,35]. Overall, the effects were sustained at 6-12 months [29,30].

Physiological and neurobiological benefits: Five studies reported physiological and neurobiological changes associated with Vipassana meditation (VM). VM altered brain topology, with Lardone et al. reporting a higher hippocampal degree (p = 0.009) [33]. Changes in event-related potentials (ERP) and heart rate variability (HRV) were noted by Delgado-Pastor et al., with increased P3b and ratio of low-frequency to high-frequency (LF/HF ratio) (p < 0.05) [37]. Krygier et al. observed improved interoception (p < 0.05) [31], while Solomonova et al. found lower occipital sleep spindles (p < 0.05) [36]. In the context of migraine, Goyal et al. reported a reduced frequency and burden by 2.7 days (95% CI -4.3 to -1.3) [28], and Rao et al. noted stress reduction alongside migraine benefits [30].

Behavioural and other benefits: Three studies examined behavioural benefits. Preferences were higher for Vipassana (Burke et al.: p < 0.001) [32]; EF improved long-term (Lukseng et al.: p < 0.05) [34]; memory consolidation patterns differed (Solomonova et al.: spindles vs. REM, p < 0.05) [36]. Reduced arrhythmia and a survival trend in heart failure were also noted (Aditee et al.: p = 0.045 AF) [27].

Subgroup and Sensitivity Analyses

Subgroups by experience: Long-term/experienced individuals showed stronger EF/neuroplasticity [34,35]. Retreat intensity: 10-day+ retreats reduced burden [29,30]. Sensitivity (excluding high bias): Consistent psychological and physiological benefits. To further elucidate these findings, Table 2 provides a domain-specific synthesis of outcomes and effect sizes from the included studies, highlighting moderators, such as retreat intensity and participant experience.

**Table 2:** Summary of Key Outcomes and Effect Sizes PSS: Perceived Stress Scale, FFMQ: Five Facet Mindfulness Questionnaire, PANAS: Positive and Negative Affect Schedule, HRV: heart rate variability, ERP: event-related potential, LF/HF: low frequency/high frequency (ratio in HRV analysis), CI: confidence interval, P3b: P300 event-related potential Subcomponent (late positive component).

Domain	Outcome Measure	Number of Studies	Effect Size Range/Key Findings	Moderators/Notes
Psychological	Stress/anxiety reduction (e.g., PSS, anxiety scales)	4 [28,29,30,34]	d=0.79 (moderate-large); p<0.05 in all; sustained at 6-12 months	Stronger in retreats; novices benefited more short term
Psychological	Mindfulness/well-being increase (e.g., FFMQ, PANAS)	3 [29,34,35]	d=0.68-0.75 (moderate); p<0.05 to 0.001; mediated by non-attachment	Experience level: stronger in mixed groups
Physiological/neurobiological	Migraine frequency/burden reduction	2 [28,30]	-2.7 days/month (95% CI -4.3 to -1.3); p<0.05	Retreat intensity: 10-day+ programs
Physiological/neurobiological	HRV/ERP/interoception improvements (e.g., LF/HF ratio, P3b)	3 [27,31,37]	p<0.05; reduced arrhythmias (p=0.045)	Lab-based; experienced practitioners
Physiological/neurobiological	Brain topology/sleep spindles (e.g., hippocampal degree, occipital spindles)	2 [33,36]	Higher degree (p=0.009); lower spindles (p<0.05)	Long-term practice
Behavioral	Executive function/memory consolidation (e.g., working memory, procedural tasks)	3 [32,34,36]	p<0.05 long-term; preferences higher (p<0.001)	Intensity: retreats; null short-term in novices

Quality Assessment

Risk of bias was assessed using the Cochrane RoB 2 tool for RCTs (n = 3) and the Newcastle-Ottawa Scale (NOS) for observational studies (n = 8) (Table 3).

**Table 3:** Risk of Bias Assessment Summary NOS Star Ratings: ★ = 1 point; Total score out of 9 (≥8 = Low risk, 6-7 = Moderate risk, ≤5 = High risk). RoB 2 Domains: Low = Low risk; Some concerns = Some concerns; High = High risk. "-" indicates not applicable for NOS-assessed studies. RoB 2: Risk of Bias 2 (Cochrane tool for randomised controlled trials), NOS: Newcastle-Ottawa Scale (tool for assessing quality of non-randomized studies), RCT: randomised controlled trial.

Study	Design	Selection/Randomization	Comparability/Deviations	Outcome/Missing Data	Measurement	Selection	Overall Risk/Score
Aditee et al. [27]	Single-arm	★★	★	★★	-	-	High
Rao et al. [28]	RCT	High	High	Some concerns	Low	Low	High
Szekeres et al. [29]	RCT	Some concerns	High	High	Low	Low	High
Goyal et al. [30]	RCT	High	High	Some concerns	Low	Low	High
Krygier et al. [31]	Observational	★★★	★	★★	-	-	Moderate
Burke et al. [32]	Observational	★★★	★	★★	-	-	Moderate
Lardone et al. [33]	Observational	★★★★	★★	★★★	-	-	Low
Lukseng et al. [34]	Observational	★★★	★	★★	-	-	Moderate
Montero-Marín et al. [35]	Single-arm	★★★	★★	★★	-	-	Moderate
Solomonova et al. [36]	Pilot	★★★	★	★★★	-	-	Moderate
Delgado-Pastor et al. [37]	Observational	★★	★	★★	-	-	High

**Figure 2:**
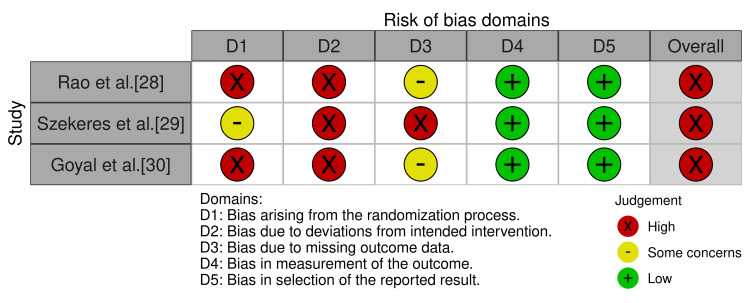
Traffic Light Plot of Risk of Bias Assessments for RCTs RCT: randomised controlled trial.

**Figure 3:**
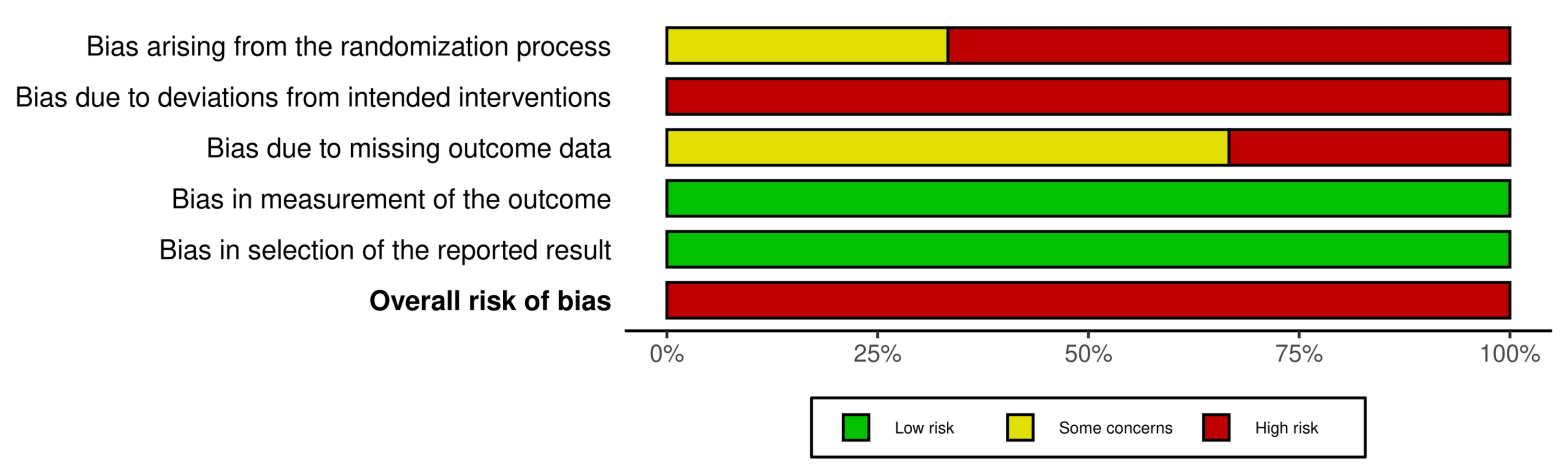
Summary Plot of Risk of Bias Assessments for RCTs RCT: randomised controlled trial.

**Figure 4:**
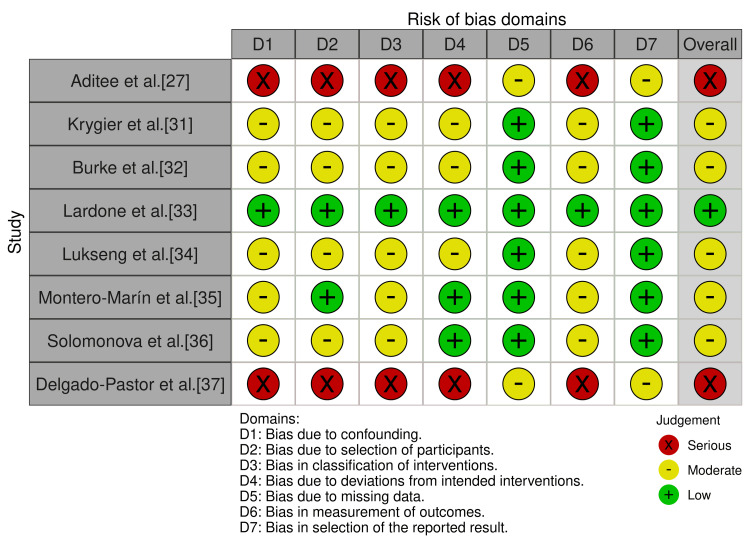
Traffic Light Plot of Risk of Bias Assessments Across Non-RCTs RCT: randomised controlled trial.

**Figure 5:**
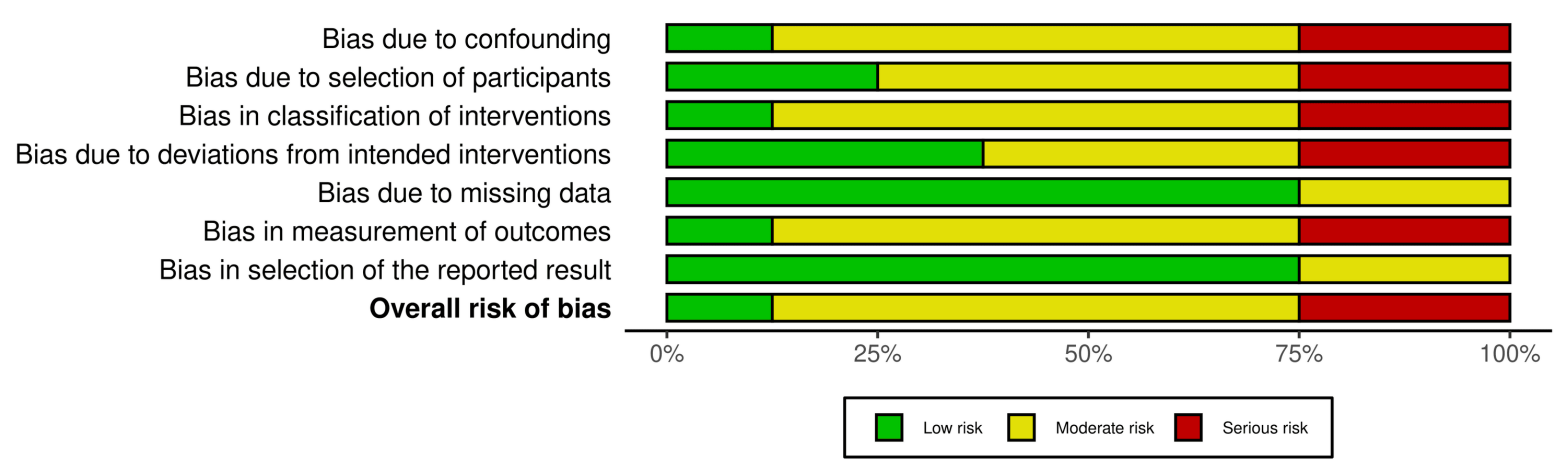
Summary Plot of Risk of Bias Assessments Across Non-RCTs RCT: randomised controlled trial.

Among the RCTs, the risk was high in all three owing to inadequate randomisation (n = 2), blinding (n = 3), and small sample sizes. For observational studies, NOS scores ranged from 5/9 to 7/9 (mean 6.5/9), with one low risk (≥8/9), four moderate risk (6-7/9), and three high risk (≤5/9). Common strengths included validated outcome measures (n = 9) and low attrition (n = 7). Weaknesses included small samples (n = 8; median N = 40), reliance on self-report (n = 6), lack of blinding (n = 10), and inadequate confounder control (n = 5) (Figures 2-5). 

No adverse events were reported in any of these studies. Heterogeneity precluded meta-analysis; narrative synthesis showed consistent positive effects, stronger with longer practice/retreats.

Discussion

The findings from the 11 included studies suggest that Vipassana meditation is associated with a range of health benefits across the physical, mental, emotional, and psychosocial domains. Physically, reductions in migraine frequency and burden were observed in two studies [28,30], with a decrease of 2.7 migraine days per 28 days and associated medication use, along with trends towards fewer arrhythmias in patients with heart failure [27]. Consistent improvements in mindfulness, executive function (e.g., working memory and cognitive flexibility), and perceived stress were reported in six studies, with effect sizes ranging from moderate to small. Emotionally, enhancements in self-kindness and positive affect, as well as reductions in anxiety and pain catastrophising, emerged in four studies, although some gains diminished at follow-up. Psychosocial benefits included altered brain topology (higher hippocampal degree) [33], improved interoception [31], modified procedural memory consolidation patterns [36], and higher preference for Vipassana over other techniques [32]. Overall, trends favoured long-term or intensive practice (e.g., retreats) for sustained effects, but null findings were noted in areas such as short-term executive functioning gains and complete retention of benefits at follow-up [29,34].

These results align with those of prior systematic reviews on Vipassana and related mindfulness interventions, although discrepancies exist because of methodological variations. Chiesa et al.'s review of seven early studies reported preliminary neurobiological changes (e.g., prefrontal activation) and clinical reductions in substance abuse among incarcerated populations [20], consistent with the current review's findings on mental and psychosocial benefits, but limited by poor quality and small samples, issues persisting in the post-2010 evidence. Auty et al.'s meta-analysis of 10 studies on mindfulness in prisons found small improvements in psychological well-being (Cohen's d = 0.46) [38], mirroring the moderate effects on stress and mindfulness observed here (e.g., d = 0.79 in Szekeres et al.) [29], yet highlighting heterogeneity in designs, which may explain why the current review's observational studies reported stronger neurobiological associations than those of clinical RCTs. Kumar et al.'s comprehensive review emphasised Vipassana's role in reducing stress and enhancing cognitive function [39], agreeing with the mental benefits observed (e.g., executive function in Lukseng et al.) [34], but differed in scope by including pre-2010 data and broader mindfulness practices, potentially overestimating effects compared to the more focused, recent evidence presented in this study. These differences may stem from the inclusion criteria and study quality, with earlier reviews incorporating lower-quality evidence, leading to more cautious conclusions. The focus of post-2010 empirical studies has captured recent advancements, such as neuroimaging and clinical applications [30,33]. However, its limitations are notable: heterogeneity in designs, populations, and outcomes precluded meta-analyses, relying on narrative synthesis potentially subject to interpretation bias. Publication bias may be present, as negative or null results were underreported, and English-language restriction could exclude relevant non-English studies. Small sample sizes (median N = 40) and high or moderate bias in most studies (e.g., lack of randomisation in 8/11 and no blinding in 10/11) limit confidence in the findings. Attrition in longer-term studies (e.g., 22% in Goyal et al.) [30] and reliance on self-reported outcomes (6/11 studies) introduced further risks of bias.

Clinically, evidence indicates that Vipassana may be a low-cost, accessible intervention for reducing stress and improving well-being, particularly in retreat formats for clinical groups such as migraine sufferers or stressed adults, where effects were sustained for up to 12 months [29,30]. Practically, it could complement conventional treatments in mental health or chronic pain management, especially for those open to intensive practice, given their associations with enhanced mindfulness and emotional regulation. Future research should prioritise larger, adequately powered RCTs with active controls, standardised outcome measures (e.g., validated scales, such as the FFMQ for mindfulness), and objective biomarkers (e.g., HRV and neuroimaging) to elucidate the underlying mechanisms. Longitudinal designs assessing long-term effects (>12 months) and adverse events are needed, as are studies exploring moderators such as experience level or dosage. Investigations in diverse populations (e.g., non-Western, paediatric) and comparative trials with other mindfulness interventions would strengthen the evidence base.

Limitations

This review is constrained by the small sample sizes in included studies, moderate to high bias risks, design heterogeneity, and lack of adverse event reporting. The narrative synthesis limits quantitative precision, and the focus on English-language publications may introduce bias. Additionally, a qualitative narrative synthesis was chosen over quantitative meta-analysis due to high heterogeneity in study designs, outcomes, and populations, small sample sizes precluding pooling, and insufficient comparable data for effect sizes.

## Conclusions

This systematic review presents moderate evidence supporting the benefits of Vipassana meditation on physical, psychological, emotional, and psychosocial health. The findings showed reductions in stress, anxiety, and migraine burden, with improvements in mindfulness, executive function, interoception, and neurobiological markers, including hippocampal topology and heart rate variability. The effects appeared to be intensity-dependent, with intensive retreats producing sustained improvements among experienced practitioners. However, methodological constraints, such as small sample sizes, high bias risk, and heterogeneity, limited generalisability and precluded meta-analysis. Clinically, Vipassana has emerged as a promising adjunctive intervention for chronic pain and stress-related disorders, potentially integrating into therapeutic programmes. Its secular nature bridges ancient practices with modern healthcare needs, addressing mental health challenges amid ongoing stressors. Despite these benefits, gaps remain in the understanding of long-term outcomes in diverse populations. Future randomised controlled trials with larger samples and objective measures are essential to confirm the efficacy of Vipassana. Advancing rigorous research can harness Vipassana's potential to promote holistic health and societal harmony.
